# A Case of Canine Sinonasal *Aspergillus fumigatus* Infection Associated With Intracranial Extension and Temporal Myositis

**DOI:** 10.1002/vms3.70188

**Published:** 2024-12-30

**Authors:** Sarah E. Cox, Giulia Cattaneo, Oliver Russell, Ombeline McGregor, Andre Kortum

**Affiliations:** ^1^ Department of Veterinary Medicine University of Cambridge Cambridge UK

**Keywords:** computed tomography, fungal, magnetic resonance imaging

## Abstract

A 12‐year‐old terrier was referred for investigation of a 4‐month history of coughing, sneezing and nasal discharge. Clinical findings were consistent with sinonasal *Aspergillus fumigatus* infection with evidence of intracranial extension on computed tomography. Endoscopic debridement followed by topical clotrimazole and systemic antifungal therapy resulted in clinical improvement. Magnetic resonance imaging after 4 weeks showed reduced intracranial disease but demonstrated evidence of temporal myositis. Repeated debridement and topical treatment were performed at 4 and 8 weeks in conjunction with long‐term voriconazole therapy. Further interventions were declined; the dog remains clinically well after 4 months with unilateral nasal discharge. As in human patients, invasive subtypes of sinonasal aspergillosis may also occur in dogs and be associated with poorer response to treatment.

## Graphical abstract

A 12‐year‐old terrier with sinonasal *Aspergillus fumigatus* infection had evidence of intracranial extension on computed tomography and was treated with endoscopic debridement, topical clotrimazole and systemic antifungal therapy. Magnetic resonance imaging after 4 weeks showed reduced intracranial disease but demonstrated evidence of temporal myositis; repeated debridement and topical treatment were performed in conjunction with long‐term systemic voriconazole therapy. As in human patients, invasive subtypes of sinonasal aspergillosis may also occur in dogs and be associated with poorer response to treatment.

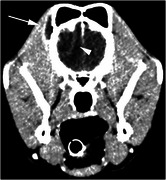

## 1 Introduction

Canine sinonasal aspergillosis (SNA) is a chronic disease of the nasal cavity and/or frontal sinus typically caused by the saprophytic fungus, *Aspergillus fumigatus*. In human patients, the disease is classified into invasive and non‐invasive subtypes, but canine SNA is primarily considered to be non‐invasive (Day 2009; Peeters, Day, and Clercx 2005). Nasal turbinate destruction and lysis of adjacent bone commonly occur in canine SNA, but, despite this aggressive behaviour, infiltration into the cranium or surrounding musculature is exceedingly rare (Prior et al. 2024).

We present the diagnosis and management of a case of canine SNA with extension of the pathology into the cranial vault and temporal musculature. To the authors’ knowledge, this is an unusual presentation of canine SNA and resembles invasive fungal rhinosinusitis in people.

## 2 Case History

A 12‐year‐old, 11‐kg male neutered terrier was referred for investigation of a 4‐month history of coughing, sneezing and nasal discharge. The dog had no other pertinent medical history and no evidence of immunocompromise. Thoracic radiography and echocardiography prior to referral were unremarkable, and blind broncho‐alveolar lavage documented mild neutrophilic inflammation with no growth on aerobic bacterial culture or direct selective fungal culture. Previous treatment with non‐steroidal anti‐inflammatories, doxycycline and omeprazole had not improved clinical signs. The patient had also received a tapering prednisolone course (starting dose 0.45 mg/kg PO q 12 h) for 1.5 months prior to referral.

At presentation, physical and neurological examinations were unremarkable other than a right‐sided mucoid nasal discharge. Haematology demonstrated a normocytic, normochromic non‐regenerative anaemia (packed cell volume 35%, reference range 37–55; reticulocytes 56 × 10^9^/L, reference range 0–70), thrombocytosis (platelets 711 × 10^9^/L, reference range 175–500) and mature neutrophilia (white blood cells 20.06 × 10^9^/L, reference range 6–17; neutrophils 16.78 × 10^9^/L, reference range 3–11.5) consistent with inflammation. Serum biochemistry and coagulation assays (prothrombin time and activated partial thromboplastin time) documented no significant abnormalities, and *Angiostrongylus vasorum* antigen testing (Idexx Angio Detect) was negative.

**FIGURE 1:**
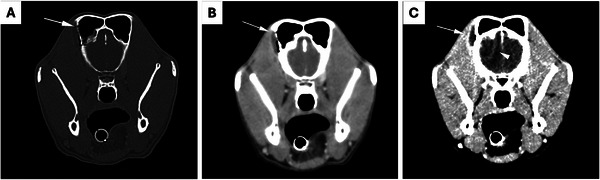
Transverse post‐contrast CT images, right is to the left of the images. (A) In a bone window; multifocal lysis of the frontal bone is visible adjacent to the fluid pocket (white arrow). (B) In a soft tissue window; a pocket of fluid‐attenuating material and gas is visible within the soft tissues immediately adjacent to the right frontal sinus, delineated by a fine, contrast‐enhancing rim (white arrow). (C) In a brain window; a pocket of fluid‐attenuating material as well as a small volume of gas is visible within the soft tissues immediately lateral to the right frontal bone, delineated by a fine contrast‐enhancing rim (white arrow). A rounded intracranial mass is visible ventromedial to the right frontal sinus, delineated by a fine, contrast‐enhancing rim (white arrowhead).

Computed tomography (CT; Toshiba Aquilion 16‐slice helical scanner) demonstrated diffuse turbinate loss in the right nasal cavity and fluid‐attenuating material within the right medial nasal cavity and maxillary and frontal sinuses. There was multifocal pinpoint lysis of the frontal bone through which fluid‐attenuating material and gas extended into the surrounding soft tissues (Figure 1A–C). At the periphery of the rostral right frontal lobe, there was a rounded non‐contrast‐enhancing region delineated by a contrast‐enhancing rim, consistent with an intracranial, extra‐axial fungal abscess (Figure 1C). There was evidence of mass effect alongside diffuse white matter tract hypoattenuation consistent with parenchymal vasogenic oedema.

**FIGURE 2:**
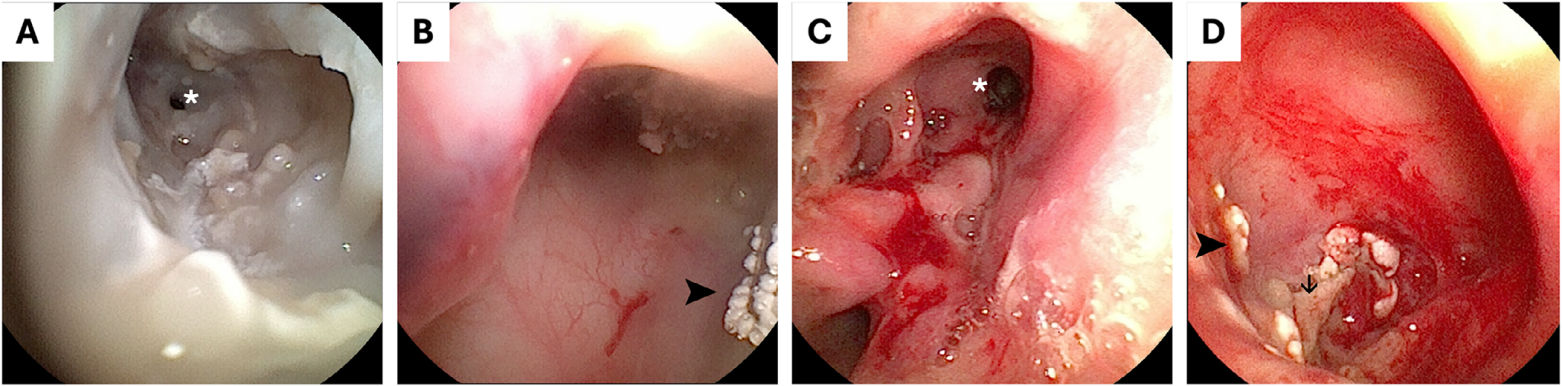
Videoendoscopic footage of the right nasal cavity and right frontal sinus. The right nasal cavity (A) and right frontal sinus (B) prior to treatment; the nasofrontal ostium is enlarged due to bony lysis (white asterisk), and there are a small number of white adhered fungal plaques (black arrowhead) in the right frontal sinus. The right nasal cavity (C) and right frontal sinus (D) 4 weeks after initial treatment; the nasofrontal ostium remains enlarged (white asterisk), and there are relatively small number of adhered white fungal plaques (black arrowheads) amongst regions of denuded bone (black arrow) in the right frontal sinus.

Anterograde rhinoscopy (Flexible Intubation Videoendoscope S5.3; Karl Storz SE & Co. KG) demonstrated mucosal erosion with copious purulent discharge within the right nasal cavity (Figure 2A). The right frontal sinus, accessed via the nasofrontal ostium, contained adhered fungal plaques (Figure 2B) which were sampled for Panfungal PCR before endoscopic debridement. A 10F Foley catheter was inserted over a guidewire into the right frontal sinus, through which 20 mL of 1% clotrimazole solution (Canesten, Bayer) was instilled, followed by 40 mL of 1% clotrimazole cream (Canesten, Bayer); the patient was placed in dorsal recumbency for 10 min prior to recovery to ensure even distribution. The dog was discharged on fluconazole (10 mg/kg PO q 12 h; Ranbaxy UK Ltd.) due to its ability to cross the blood–brain barrier (Hector 2005).

**FIGURE 3:**
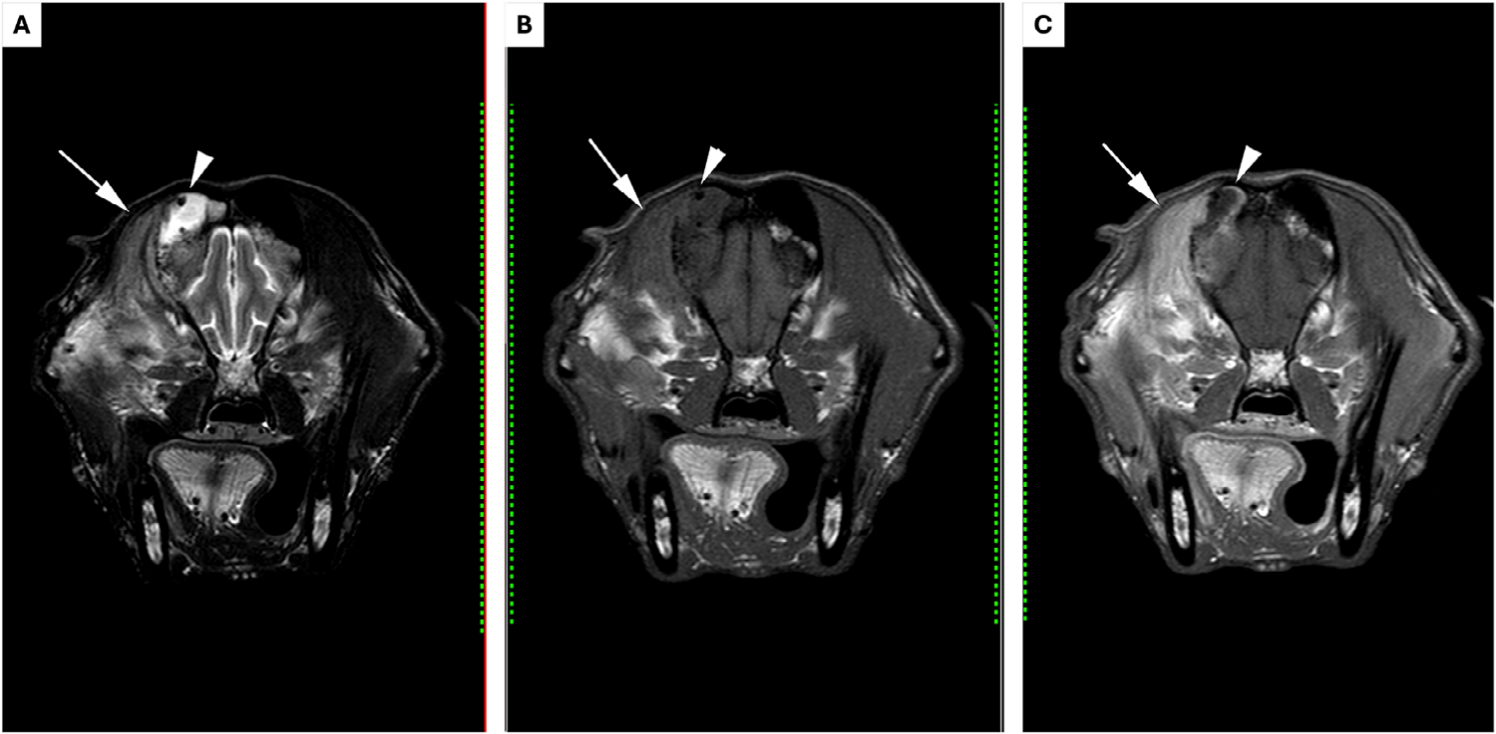
Transverse MRI images, with a T2w sequence (A), a T1w pre‐contrast sequence (B) and T1w post‐contrast sequence (C). Right is to the left of each image. The right frontal sinus is filled with T2w‐hyperintense, T1w‐hypointense, non‐contrast‐enhancing material along with rounded regions of signal void consistent with gas (white arrowhead). There is adjacent marked thickening and contrast enhancement of the mucosal lining of the frontal sinus and multifocal discontinuity of the frontal bone. There is marked right temporal muscle atrophy, with increased T2w/ T1w hyperintensity and contrast enhancement of the muscle when compared to the contralateral side (white arrow).

**FIGURE 4:**
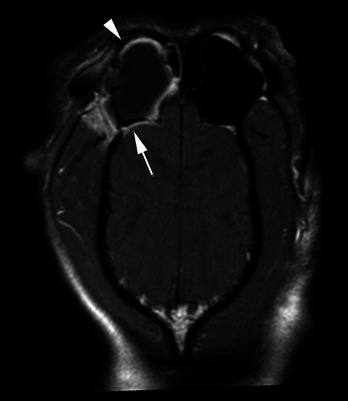
Dorsal MRI image (T1w post‐contrast). Right is to the left of the image. A large volume of T1w‐hypointense, non‐contrast‐enhancing material fills the dependent aspect of the right frontal sinus (the patient was placed in dorsal recumbency). This is associated with marked thickening and contrast enhancement of the mucosal lining of the frontal sinus (white arrowhead). There is adjacent focal marked meningeal thickening and contrast enhancement (white arrow).

The dog's demeanour and sneezing improved; however, the right‐sided nasal discharge persisted. A cluster of three generalised seizures occurred 4 days after treatment which was well controlled with oral phenobarbitone (2.5 mg/kg PO q 12 h; Epiphen, Vetoquinol UK Ltd), with only one subsequent seizure. Serum phenobarbitone concentration was monitored closely due to the inhibitory action of azoles on cytochrome P450 enzymes (Hector 2005), and the dose was adjusted as required. On reassessment at 4 weeks, haematology was unremarkable, but biochemistry demonstrated increased ALP (317 IU/L, reference range 26–107), which can be observed following treatment with both fluconazole and phenobarbitone in dogs (Berlin et al. 2024; Müller et al. 2000). On rhinoscopy, adhered fungal plaques were noted only within the right frontal sinus (Figure 2C,D). Complete debridement and topical treatment were repeated. In light of the previous intracranial changes, magnetic resonance imaging (MRI; 1.5 Tesla scanner; Achieva, Phillips Electronics UK Limited Ascent, Farnborough, UK) was performed. This demonstrated diffuse right temporal muscle hyperintensity and atrophy consistent with chronic myositis. The fluid material adjacent to the frontal bone was smaller in volume compared to previous imaging (Figure 3A–C). The intracranial fluid‐filled mass was no longer visible, but there was focal meningeal thickening and hyperintensity consistent with meningitis (Figure 4). Results of the previous Panfungal PCR confirmed *A. fumigatus*, and voriconazole (5 mg/kg PO q 12 h; Aristo Pharma Ltd.) was prescribed in place of fluconazole due to reported intrinsic resistance of *A. fumigatus* to the latter (Hector 2005). A short course of amoxicillin/ clavulanic acid (24 mg/kg PO q 12 h; Clavaseptin, Vetoquinol UK Ltd) was prescribed due to concern for secondary bacterial myositis.

**FIGURE 5:**
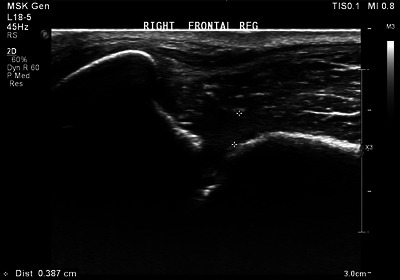
Ultrasound image of the right frontal region. There is focal discontinuity of the right frontal bone with an elongated accumulation of hypoechoic tissue overlying it (outlined by the callipers). This tissue lacks the normal fibrillar appearance of the muscle visible superficial to it.

At eight weeks a further improvement in clinical signs was reported, but mild unilateral nasal discharge and occasional sneezing persisted. Right frontal sinuscopy demonstrated a single fungal plaque amongst regions of exposed bone that were mobile with external pressure. Mucosal biopsies were taken before debridement and topical clotrimazole treatment as previously described. Focal temporal ultrasound showed fenestration of the frontal bone and rostral calvarium with an elongated region of superficial hypoechoic tissue (Figure 5). Histopathology of the frontal sinus mucosa demonstrated pyogranulomatous sinusitis with numerous intralesional, septate fungal hyphae approximately 5 microns in diameter with parallel walls and acute angle dichotomous branching; however, insufficient normal mucosa was present to definitively confirm invasion.

Due to the refractory nature of the disease and the suspicion of concurrent osteomyelitis with extension into the temporal soft tissues, surgical debridement was recommended; this option was declined on financial grounds. As such, an extended course of oral voriconazole was recommended, and a guarded prognosis was advised. On follow‐up after 4 months, the dog was reported to have remained clinically well aside from persistent, mild unilateral nasal discharge and occasional sneezing.

## 3 Discussion

Canine SNA is generally regarded as a chronic, non‐invasive disease (Peeters, Day, and Clercx 2005), but lysis of the cribriform plate is observed on CT in 1% of cases (Prior et al. 2024). Histopathology of canine SNA typically demonstrates fungal hyphae overlying the mucosa (Peeters, Day, and Clercx 2005); tissue destruction is thought to result from the interaction between the *A. fumigatus* biofilm and the mucosal immune system rather than invasion (Valdes et al. 2020). Involvement of adjacent tissues is rarely described in immunocompetent dogs, although ventricular pneumocephalus, cervical subarachnoid pneumorrhachis and meningoencephalitis were reported following surgical management of chronic fungal rhinitis in one dog (Launcelott et al. 2016).

Human fungal rhinosinusitis has a broader clinical spectrum, with non‐invasive disease subdivided into saprophytic fungal infestation, fungal ball and allergic fungal rhinosinusitis and invasive disease comprising acute, chronic and granulomatous forms (Chakrabarti et al. 2009). Canine SNA parallels non‐invasive fungal rhinosinusitis in humans, with densely matted fungal hyphae and inflammatory cells resembling fungal balls within the airspaces. However, the clinical features of this case bear more similarity to invasive fungal rhinosinusitis which is associated with more aggressive behaviour including orbito‐cerebral infiltration (Chakrabarti et al. 2009).

Aspergillosis of the central nervous system (CNS) in human patients is widely regarded as an invasive disease resulting from haematogenous or contiguous spread; in immunocompetent patients, it is commonly associated with spread from the paranasal sinuses (Meena et al. 2021). Whilst there is some inconsistency in the terminology describing CNS aspergillosis in dogs, it is likely to be a similarly invasive process, and a single case associated with sinusitis has been reported in a dog that received immunosuppressive chemotherapy (Taylor et al. 2015). Invasion is defined histopathologically in human patients by the presence of fungal hyphae within the deeper tissues (Chakrabarti et al. 2009), and whilst CT findings are insensitive and non‐specific, observed patterns of aspergillosis within cerebral tissue may include ring‐enhancing lesions and dural enhancement, as demonstrated in this case (Ashdown, Tien, and Felsberg 1994; El Hasbani et al. 2019). The management of invasive fungal rhinosinusitis in humans comprises surgical debridement with systemic anti‐fungal agents (Deutsch, Whittaker, and Prasad 2019).

Compared to human invasive fungal rhinosinusitis, there is no consensus on the treatment of canine SNA; however, debridement and topical anti‐fungal treatment are widely regarded as superior to systemic therapy. Given the lack of reported adverse neurological events in studies evaluating topical clotrimazole application in dogs with cribriform plate lysis, this was deemed a reasonable treatment option (Belda, Petrovitch, and Mathews 2018; Stanton et al. 2018). Moreover, considering the delay prior to development of seizures, it was considered likely that these related to the underlying intracranial pathology, but an adverse reaction to topical treatment cannot be entirely excluded. Limited studies have evaluated the use of oral voriconazole in canine SNA, but acceptable outcomes have been reported alongside surgical debridement (Bray et al. 2020).

Whilst the initial diagnosis of fungal rhinosinusitis based on clinical signs, the visualisation of fungal plaques and compatible CT findings was similar to that previously described, the lack of histopathological confirmation of SNA until late in the disease course is a limitation of this case. Laboratory findings in canine SNA are variable and non‐specific, although mild neutrophilia is commonly observed and anaemia is reported (Prior et al. 2024). Whilst other differentials for an intra‐cranial, extra‐axial mass were considered at the time, the positive response following debridement and anti‐fungal therapy would support the primary differential of a fungal abscess. The observed myositis could have resulted from inflammation secondary to infection rather than direct fungal invasion, as is proposed for SNA‐associated tissue damage (Crum‐Cianflone 2008; Valdes et al. 2020). Myositis unrelated to the fungal infection was considered unlikely, but attempts to demonstrate fungal hyphae within the lesion were limited by the low cellularity of fine needle aspirate cytology. In future cases where an invasive subtype of SNA is suspected, early histopathological confirmation from sinonasal and myositic lesions should be considered to confirm direct involvement of the organism.

This report describes the presentation and progression of an unusually aggressive case of canine SNA resembling invasive fungal rhinosinusitis in humans. This could suggest that SNA in dogs is in fact a spectrum of diseases, rather than one distinct entity, and that evidence of invasive disease may confer a negative prognosis with more aggressive behaviour, as in humans with invasive fungal rhinosinusitis.

## Author Contributions

**Sarah E. Cox**: writing–original draft (lead), writing–review and editing (equal). **Giulia Cattaneo**: writing–review and editing (equal). **Oliver Russell**: writing–review and editing (equal). **Ombeline McGregor**: writing–original draft (equal), writing–review and editing (equal). **Andre Kortum**: supervision (lead), writing–review and editing (equal).

## Ethics Statement

The authors confirm that the ethical policies of the journal have been adhered to. No ethical approval was required as no original research data were collected. Informed consent was gained for all clinical procedures.

## Conflicts of Interest

The authors declare no conflicts of interest.

### Peer Review

The peer review history for this article is available at https://publons.com/publon/10.1002/vms3.70188.

## Data Availability

The data that support the findings of this study are available from the corresponding author upon reasonable request.

## References

[vms370188-bib-0001] Ashdown, B. C. , R. D. Tien , and G. J. Felsberg . 1994. “Aspergillosis of the Brain and Paranasal Sinuses in Immunocompromised Patients: CT and MR Imaging Findings.” American Journal of Roentgenology 162: 155–159. 8273655 10.2214/ajr.162.1.8273655

[vms370188-bib-0002] Belda, B. , N. Petrovitch , and K. G. Mathews . 2018. “Sinonasal Aspergillosis: Outcome After Topical Treatment in Dogs With Cribriform Plate Lysis.” Journal of Veterinary Internal Medicine 32: 1353–1358. 29957889 10.1111/jvim.15219 PMC6060319

[vms370188-bib-0003] Berlin, D. , J. A. Jaffey , C. Bolch , et al. 2024. “Serial Evaluation of Liver Enzyme Activities in Dogs With Pulmonary Coccidioidomycosis Administered Per Os Fluconazole.” Frontiers in Veterinary Science 11: 1402572. 39315088 10.3389/fvets.2024.1402572 PMC11417468

[vms370188-bib-0004] Bray, R. N. , C. L. Raghu , A. S. Leuin , et al. 2020. “Oral Administration of Voriconazole With Surgical Fungal Plaque Debridement for the Treatment of Sinonasal Aspergillosis With Cribriform Plate Lysis in Three Dogs.” Journal of the American Veterinary Medical Association 256: 111–116. 31841098 10.2460/javma.256.1.111

[vms370188-bib-0005] Chakrabarti, A. , D. W. Denning , B. J. Ferguson , et al. 2009. “Fungal Rhinosinusitis: A Categorization and Definitional Schema Addressing Current Controversies.” The Laryngoscope 119: 1809–1818. 19544383 10.1002/lary.20520 PMC2741302

[vms370188-bib-0006] Crum‐Cianflone, N. F. 2008. “Bacterial, Fungal, Parasitic, and Viral Myositis.” Clinical Microbiology Reviews 21: 473–494. 18625683 10.1128/CMR.00001-08 PMC2493084

[vms370188-bib-0007] Day, M. J. 2009. “Canine Sino‐Nasal Aspergillosis: Parallels With Human Disease.” Medical Mycology 47: S315–S323. 18608893 10.1080/13693780802056038

[vms370188-bib-0008] Deutsch, P. G. , J. Whittaker , and S. Prasad . 2019. “Invasive and Non‐Invasive Fungal Rhinosinusitis—A Review and Update of the Evidence.” Medicina 55: 319. 31261788 10.3390/medicina55070319 PMC6681352

[vms370188-bib-0009] El Hasbani, G. , J. Chirayil , S. Nithisoontorn , et al. 2019. “Cerebral Aspergillosis Presenting as a Space Occupying Lesion in an Immunocompetent Individual.” Medical Mycology Case Reports 25: 45–48. 31453078 10.1016/j.mmcr.2019.07.011 PMC6700407

[vms370188-bib-0010] Hector, R. F. 2005. “An Overview of Antifungal Drugs and Their Use for Treatment of Deep and Superficial Mycoses in Animals.” Clinical Techniques in Small Animal Practice 20: 240–249. 16317914 10.1053/j.ctsap.2005.07.005

[vms370188-bib-0011] Launcelott, Z. A. , M. P. Palmisano , J. D. Stefanacci , et al. 2016. “Ventricular Pneumocephalus, Cervical Subarachnoid Pneumorrhachis, and Meningoencephalitis in a Dog Following Rhinotomy for Chronic Fungal Rhinitis.” Journal of the American Veterinary Medical Association 248: 430–435. 26829276 10.2460/javma.248.4.430

[vms370188-bib-0012] Meena, D. S. , D. Kumar , G. K. Bohra , et al. 2021. “Clinical Manifestations, Diagnosis, and Treatment Outcome of CNS Aspergillosis: A Systematic Review of 235 Cases.” Infectious Diseases Now 51: 654–660. 33964485 10.1016/j.idnow.2021.04.002

[vms370188-bib-0013] Müller, P. B. , J. Taboada , G. Hosgood , et al. 2000. “Effects of Long‐Term Phenobarbital Treatment on the Liver in Dogs.” Journal of Veterinary Internal Medicine 14: 165–171. 10772488 10.1111/j.1939-1676.2000.tb02231.x PMC7197517

[vms370188-bib-0014] Peeters, D. , M. J. Day , and C. Clercx . 2005. “An Immunohistochemical Study of Canine Nasal Aspergillosis.” Journal of Comparative Pathology 132: 283–288. 15893986 10.1016/j.jcpa.2004.11.002

[vms370188-bib-0015] Prior, C. , H. Swales , M. Sharman , et al. 2024. “Diagnostic Findings in Sinonasal Aspergillosis in Dogs in the United Kingdom: 475 Cases (2011–2021).” Journal of Small Animal Practice 65: 622–630. 38679786 10.1111/jsap.13736

[vms370188-bib-0016] Stanton, J. A. , M. L. Miller , P. Johnson , et al. 2018. “Treatment of Canine Sinonasal Aspergillosis With Clotrimazole Infusion in Patients With Cribriform Plate Lysis.” The Journal of Small Animal Practice 59: 411–414. 29602218 10.1111/jsap.12835

[vms370188-bib-0017] Taylor, A. R. , B. D. Young , G. J. Levine , et al. 2015. “Clinical Features and Magnetic Resonance Imaging Findings in 7 Dogs With Central Nervous System Aspergillosis.” Journal of Veterinary Internal Medicine 29: 1556–1563. 26473515 10.1111/jvim.13648 PMC4895661

[vms370188-bib-0018] Valdes, I. D. , A. B. P. Hart de Ruijter , C. J. Torres , et al. 2020. “The Sino‐Nasal Warzone: Transcriptomic and Genomic Studies on Sino‐Nasal Aspergillosis in Dogs.” npj Biofilms and Microbiomes 6: 51. 33184275 10.1038/s41522-020-00163-7 PMC7665010

